# Presence of alarm symptoms at coeliac disease diagnosis is not associated with poorer long-term treatment outcomes

**DOI:** 10.1097/MEG.0000000000003073

**Published:** 2025-09-22

**Authors:** Eneli Katunin, Camilla Pasternack, Kalle Kurppa, Teea Salmi, Heini Huhtala, Katri Kaukinen, Rakel Nurmi

**Affiliations:** a Celiac Disease Research Center, Faculty of Medicine and Health Technology, Tampere University, Tampere; b Department of Internal Medicine, Kanta-Häme Central Hospital, Hämeenlinna; c Department of Dermatology, Tampere University Hospital; d Tampere Center for Child, Adolescent, and Maternal Health Research, Tampere University and Tampere University Hospital, Wellbeing Services County of Pirkanmaa, Tampere; e The University Consortium of Seinäjoki, Seinäjoki; f Faculty of Social Sciences, Tampere University; g Department of Internal Medicine, Tampere University Hospital, Wellbeing Services County of Pirkanmaa, Tampere, Finland

**Keywords:** alarm symptoms, anaemia, coeliac disease, weight loss

## Abstract

**Objectives:**

Alarm symptoms at coeliac disease (CeD) diagnosis predict a more severe disease presentation, but the long-term implications remain unclear. We studied the prevalence of alarm symptoms at diagnosis and their association with outcomes.

**Methods:**

A mixed-method cohort study combined retrospective medical record review with data collection through patient interviews and blood sampling from 814 adult patients with CeD after a median of 9.7 years on a gluten-free diet (GFD). Validated questionnaires assessed symptoms and quality of life. Alarm symptoms included anaemia, weight loss, dysphagia, vomiting, melaena, and rectal bleeding. Patients were grouped by the presence or absence of alarm symptoms.

**Results:**

45% of the patients presented with alarm symptoms, primarily (95%) anaemia and weight loss. These patients were significantly more often female (83 vs. 71%; *P* < 0.001), had more severe clinical presentation (*P* < 0.001; reported severe symptoms 41 vs. 2%) and more advanced mucosal damage (*P* < 0.001; subtotal or total villous atrophy 72 vs. 57%) than those without these symptoms. On GFD, these patients experienced fewer persistent symptoms (asymptomatic 71 vs. 79%; *P* = 0.035) but more often had osteopenia/osteoporosis (15 vs. 9%; *P* = 0.008). The groups did not differ in the strictness of GFD, positivity of CeD autoantibodies, quality of life, fractures, or other comorbidities.

**Conclusion:**

Alarm symptoms were common at CeD diagnosis. After 9.7 years on a GFD, patients with alarm symptoms had a higher incidence of osteopenia/osteoporosis, but generally did not demonstrate poorer long-term outcomes compared to those without alarm symptoms.

## Introduction

Coeliac disease (CeD) is a common immune-mediated disorder, characterized by diverse clinical presentations, including various gastrointestinal and extraintestinal symptoms [1]. The so-called alarm symptoms, such as unintentional weight loss, anaemia, and dysphagia, may indicate untreated CeD, but they may also be signs of other intestinal disorders and even malignancies [2,3]. At present, data on the prevalence and health implications of alarm symptoms at CeD diagnosis are scarce, with the exception of some information regarding anaemia. Anaemia has been reported to be present in up to 85% of untreated patients with CeD [4–6] and to indicate severe disease presentation at diagnosis [5,7,8]. It remains, however, unclear if these findings are associated with poorer long-term treatment outcomes.

In recent years, the feasibility of serology-based ‘no-biopsy’ diagnosis, also for adult patients with CeD, has been increasingly debated [9–11]. It would be important to obtain more information on possible clinical features that still necessitate endoscopic investigations of the upper gastrointestinal tract. For example, in patients with dyspepsia, oesophagogastroduodenoscopies (OGD) are typically reserved for those presenting with alarm symptoms [12]. There is some evidence that, despite being frequently present at CeD diagnosis, alarm symptoms do not predict overrepresentation of clinically relevant findings in endoscopy [2,3]. Overall, however, there are limited data on the general health, quality of life, and prevalence of comorbidities in patients with CeD presenting with alarm symptoms.

We aimed to investigate the prevalence of alarm symptoms in untreated CeD, as well as their association with various patient-related outcomes at diagnosis and during long-term adherence to a GFD.

## Materials and methods

### Patients, study design, and ethics

The study was a mixed-method cohort study, combining retrospective medical record review with prospective data collection through patient interviews and blood sampling during follow-up visits conducted at Tampere University and Tampere University Hospital. Adult patients with a biopsy-proven CeD diagnosis were recruited between the years 2006 and 2010 through patient organization meetings, public lectures, family members, and newspaper advertisements. Exclusion criteria included age less than 18 years, unconfirmed CeD diagnosis, and lack of adequate medical data. After exclusions, the final cohort comprised 814 patients, all of whom were invited to provide comprehensive patient-related data either during a separate study visit or by phone, utilizing predefined study questions administrated by a physician or study nurse. Furthermore, validated questionnaires were used during the study visit to evaluate the severity of current gastrointestinal symptoms and health-related quality of life, and blood samples were drawn for CeD serology. In addition, relevant medical data recorded at the CeD diagnosis and during subsequent healthcare visits were retrospectively collected from the patients’ records. For the analyses, participants were further divided into two groups according to the presence or absence of alarm symptoms at diagnosis. Subgroup analyses were conducted for anaemia and weight loss.

The Regional Ethics Committee of the Tampere University Hospital District approved the study protocol and patient recruitment (Ethics committee approval R05183). All participants provided written informed consent. The principles of the Declaration of Helsinki were followed at all stages.

## Data collection

### Data at coeliac disease diagnosis

The collected data included year of and age at CeD diagnosis, sex, type, severity, and duration of the main presenting symptoms, as well as positivity for serum endomysial antibodies (EmA), tissue transglutaminase antibodies (TGA), or both. In addition, the degree of small-bowel mucosal damage was obtained from the pathology reports, which are routinely included in the patients’ medical records.

Clinical presentation was further categorized into gastrointestinal (e.g. diarrhoea, abdominal pain, bloating, constipation, and nausea), extraintestinal (e.g. dermatitis herpetiformis, infertility, arthralgia, and neurological symptoms), and asymptomatic (screen-detected). Severity of symptoms was divided into mild (occasional CeD-related symptoms), moderate (symptoms moderately affecting daily life) and severe (symptoms significantly affecting daily life), and their duration into less than 1, 1–5, 5–10, and greater than 10 years. Alarm symptoms were defined as anaemia, unintentional weight loss, dysphagia, recurrent vomiting, melaena, and rectal bleeding. Anaemia was defined as a hemoglobin level less than 134 g/L in men and less than 117 g/L in women, based on national reference values [13]. Weight loss was considered present if the patient reported unintentional loss of body weight. The severity of small-bowel mucosal lesions was further categorized into morphologically normal, partial villous atrophy (Marsh–Oberhuber IIIa) and subtotal/total villous atrophy (IIIb/c). A subgroup of patients with lesions milder than degree III was diagnosed on the basis of a combination of CeD-associated autoantibodies and histological findings, as well as special methods including determination of the intestinal transglutaminase-specific immunoglobulin A (IgA) deposits from frozen mucosal biopsies.

### Long-term follow-up data

The data collected included current age, duration and strictness of GFD, and presence of persistent CeD-related symptoms and chronic comorbidities (including osteopenia or osteoporosis and fractures). Results of a possible re-biopsy after 1 year on GFD were collected from follow-up reports. The patients, moreover, completed self-administered validated questionnaires regarding their current symptoms and quality of life during the separate study visit. In addition, a structured interview regarding potential CeD-related symptoms at the time of diagnosis was systemically conducted by a study nurse or physician to supplement the retrospective medical record data.

Comorbidities were further classified into musculoskeletal, gastrointestinal, psychiatric, and autoimmune disorders and malignancies. Basal cell carcinoma was excluded from the malignancy analyses because of its typically nonmetastatic behavior and high incidence, which could distort the overall cancer burden assessment. The severity of symptoms and the degree of small-bowel mucosal lesions were classified similarly as at diagnosis. Adherence to GFD was assessed through an interview conducted by an experienced study nurse or study physician, and further classified as strict diet, occasional lapses, or no diet. Response and adherence to GFD were also assessed indirectly by measuring CeD serology at the study visit. IgA-class EmA were measured by indirect immunofluorescence, considering titers 1 : ≥5 positive [14]. A commercial test (QUANTA Lite h-tTG IgA; INOVA Diagnostics, San Diego, California, USA) was used to measure IgA-class TGA, considering values greater than 30 U/L positive.

Symptoms and quality of life were assessed using the Gastrointestinal Symptom Rating Scale (GSRS) and the Psychological General Well-Being Questionnaire (PGWB). The GSRS evaluates overall gastrointestinal symptoms and subgroups of indigestion, diarrhoea, constipation, abdominal pain, and reflux. It contains 15 items assessed on a 7-point Likert scale, higher scores indicating more severe symptoms [15,16]. The PGWB evaluates wellbeing with 22 items (6-point Likert scale), further subclassified into anxiety, depression, wellbeing, self-control, general health, and vitality. Higher scores represent better quality of life and wellbeing [17,18].

### Statistics

Categorical variables were reported as numbers and percentages and continuous variables as medians with interquartile ranges. Cross-tabulation with *χ*^2^ or Fisher’s exact test was used to test the statistical significance for categorical variables and Mann–Whitney *U* test for continuous variables. Binary logistic regression analysis was used to adjust for sex and duration of GFD. *P* values less than 0.05 were considered statistically significant. The data were analyzed with SPSS 29 software package (IBM Corp., New York, New York, USA).

## Results

### Data at diagnosis

Overall, 364 (45%) participants presented with one or more alarm symptoms, including 204 (25%) with anaemia, 90 (11%) with weight loss, and 52 (6%) with both. Rarer alarm symptoms included recurrent vomiting (*n* = 30, 4%), rectal bleeding (*n* = 4, 0.5%), and melaena (*n* = 4, 0.5%). None of the patients had dysphagia. Patients with alarm symptoms were more often women and exhibited a higher prevalence of severe symptoms and advanced histological damage in both crude analysis and after adjusting for sex, whereas the groups did not differ in age, duration, or type of symptoms, or positivity of CeD serology (Table 1).

**Table 1.** Characteristics of 814 coeliac disease patients with or without alarm symptoms at diagnosis

Characteristics	Alarm symptoms (*n* = 364), %	No alarm symptoms (*n* = 450), %	*P* value
Females	83.0	70.9	**<0.001**
Age, median (quartiles), years	43 (43–50)	44 (33–54)	0.137
Clinical presentation			
Asymptomatic^a^	4.9	5.6	0.699
Gastrointestinal symptoms	82.7	82.0	0.797
Extraintestinal symptoms	15.4	17.6	0.408
Severity of symptoms			**<0.001** *
Mild	16.4	36.2	
Moderate	43.0	61.4	
Severe	40.6	2.4	
Duration of symptoms (years)			0.075
<1	18.3	23.0	
1–5	33.3	34.9	
5–10	9.1	11.4	
>10	39.2	30.6	
Positive CeD serology^b,c^	84.7	87.7	0.363
Small-bowel mucosal damage^d^			**<0.001** *
Normal histology^e^	2.5	3.4	
Partial villous atrophy	25.9	40.2	
Subtotal or total villous atrophy	71.7	56.5	

Bolded values denote statistical significance.

CeD, coeliac disease; IgA, immunoglobulin A.

a Screen-detected patients in at-risk groups of CeD.

b Endomysial and tissue transglutaminase antibodies.

c Data were available from 433 (53%).

d Data were available from 678 (83%) patients; data were missing for the remaining patients.

e CeD was diagnosed on the basis of a combination of serological and histological findings and special methods, such as mucosal transglutaminase-specific IgA deposits.

* *P* < 0.05 after adjusting for sex.

### Follow-up data

Median follow-up time on GFD was 9.7 years. Patients with alarm symptoms at diagnosis had longer duration of GFD and less often persistent symptoms, but a higher prevalence of osteopenia or osteoporosis than those without alarm symptoms, both in crude analysis and after adjusting for sex and duration of GFD (Table 2). The groups did not differ in reported adherence to GFD, positivity to EmA or TGA, prevalence of fractures, malignancies and other comorbidities, or degree of histological damage 1 year after diagnosis (Table 2). Nor was there any difference in GSRS and PGWB total (Table 2) or subgroup scores (data not shown).

**Table 2.** Characteristics of 814 coeliac disease patients with or without alarm symptoms on a gluten-free diet

Characteristics	Alarm symptoms (*n* = 364), %	No alarm symptoms (*n* = 450), %	*P* value
Age, median (quartiles), years	54 (45–63)	55 (44–64)	0.741
Duration of GFD, median (quartiles), years	10 (5–16)	8 (4–14)	**0.007**
Strictness of GFD			0.268
Strict diet	97.8	96.9	
Occasional lapses	1.9	3.1	
Normal gluten-containing diet	0.3	0.0	
Duodenal histology after 1 year on GFD^a^			0.093
Normal histology	53.2	63.0	
Partial villous atrophy	40.4	32.9	
Subtotal or total villous atrophy	6.4	4.2	
Positive endomysial antibodies	6.9	6.9	1.00
Positive transglutaminase antibodies	11.1	10.9	0.940
Persistent CeD-related symptoms			**0.035** *
No	78.6	70.9	
Mild	19.4	27.3	
Severe	1.9	1.8	
Chronic comorbidities			
Gastrointestinal disease	34.6	34.9	0.935
Autoimmune disease	44.0	40.4	0.313
Musculoskeletal disease	17.6	23.3	**0.046**
Osteoporosis or osteopenia	14.8	8.9	**0.008** *
Any fracture	29.5	25.8	0.248
Psychiatric disease	4.9	4.2	0.623
Malignancy	2.7	3.8	0.741
GSRS total score, median (quartiles)^b^	2.0 (1.5–2.6)	1.9 (1.5–2.5)	0.629
PGWB total, median (quartiles)^b^	106 (94–116)	106 (95–115)	0.985

Values in bold face denote statistical significance.

CeD, coeliac disease; GFD, gluten-free diet; GSRS, Gastrointestinal Symptom Rating Scale; PGWB, psychological general well-being.

a Data were available from 462 (57%).

b Data were available from 590 (73%) of patients.

* *P* < 0.05 after adjusting for sex and duration of GFD.

Altogether, 10 patients with and 18 patients without alarm symptoms at diagnosis were diagnosed with malignancies. Among those with alarm symptoms, three had breast cancer, two had prostate cancer, two had small-bowel cancer, and three had other types of cancers. Patients with small-bowel cancers experienced unintentional weight loss and recurrent vomiting. Among those without alarm symptoms, five had breast cancer, two had prostate cancer, two had colon cancer, two had thyroid cancer, two had ovarian cancer, and five had other types of cancer.

### Patients presenting with anaemia or weight loss

In subgroup analysis, patients presenting with anaemia were significantly more likely to be women, had a higher prevalence of gastrointestinal symptoms and longer duration of symptoms before diagnosis, and had more advanced histological lesions compared to those without anaemia (Supplementary Table S1, Supplemental digital content 1, https://links.lww.com/EJGH/B217). At follow-up, anaemic patients reported less often musculoskeletal diseases and a higher prevalence of osteoporosis or osteopenia (Supplementary Table S2, Supplemental digital content 2, https://links.lww.com/EJGH/B218).

Patients presenting with weight loss were less often otherwise asymptomatic, more often experienced gastrointestinal and severe symptoms, were more often seronegative, and had more advanced histological lesions compared to those without weight loss (Supplementary Table S1, Supplemental digital content 1, https://links.lww.com/EJGH/B217). At follow-up, patients with weight loss at diagnosis were older and had been on a GFD for longer (Supplementary Table S2, Supplemental digital content 2, https://links.lww.com/EJGH/B218). In addition, they had a lower rate of histological recovery after 1 year on GFD compared with patients without weight loss.

## Discussion

Alarm symptoms were common at CeD diagnosis and were present in almost half of the patients in the study cohort; however, anaemia and unintentional weight loss accounted for up to 95% of these, whereas the other alarm symptoms considered here were rare. In comparison, the prevalence of anaemia in adults with untreated CeD has varied from 12% to up to 85% according to earlier studies [3,5,8,19–21]. The wide range likely reflects geographical and genetic differences between the populations studied. For example, a prevalence of 85% was reported in India [5], whereas Western countries have generally reported figures ranging from 20 to 46% [3,6,19–21]. Other alarm symptoms have been less well studied, and data concerning their prevalence are scarce. According to the study by Maimaris *et al*., [3] the prevalence of unintentional weight loss was 23%, rectal bleeding 4%, recurrent vomiting 3%, and dysphagia less than 1%. While weight loss is a well-known symptom of CeD, the significance of the other clinical manifestations in this context is less clear. Although dysphagia and even rectal bleeding may sometimes be signs of untreated CeD [22–26], this is unlikely, and their presence should raise suspicion of some other etiology.

Patients with alarm symptoms also presented otherwise with more severe clinical features and more advanced histological disease at CeD diagnosis. When analyzed separately, the same findings were demonstrated in the anaemia subgroup, which is in line with several earlier studies indicating that patients with anaemic CeD exhibit more clinically and histologically advanced CeD [5–8]. Corresponding findings of more severe disease features were also observed in subjects with weight loss; however, while there were no differences in serology based on alarm symptoms as a whole or on anaemia, patients in the weight loss subgroup were more often seronegative. This corroborates with earlier reports by Aziz *et al*. [27] and Schiepatti *et al*. [28], as well as evidence suggesting that seronegative patients often have complicated CeD with malabsorptive symptoms [29]. Nonetheless, it should be noted that selection bias is likely, as asymptomatic seronegative individuals have no incentive to undergo OGD. Of note, female sex was also associated with alarm symptoms. A plausible explanation is the already high prevalence of anaemia among women of menstrual age, which may be exacerbated by inflammation and malabsorption related to untreated CeD [4].

After a median follow-up of approximately 10 years, the only indicator of poorer long-term health outcomes in patients presenting with alarm symptoms at CeD diagnosis was a higher prevalence of osteoporosis or osteopenia, even after adjusting for sex. No significant difference in histological recovery was observed between the groups after 1 year on GFD, but there was a trend toward poorer recovery in the group with alarm symptoms, which may partially explain the finding. Of note, despite the excellent adherence to GFD, approximately 40% of patients showed incomplete mucosal recovery after 1 year. This finding aligns with previous studies [28–30] and may reflect differences in histological classifications and definitions of mucosal normalization, as well as genetic variation across populations. In our and Norwegian studies, nearly all patients eventually achieved recovery, and slower healing had no clinical relevance [30,31]. No subsequent biopsy data were available in the present study, and further research on the association between slow mucosal healing and the prevalence of osteoporosis or osteopenia is needed. In any case, there was no significant difference in the prevalence of fractures at the time of long-term follow-up. The study groups were also comparable in terms of malignancies and health-related quality of life as measured by two validated questionnaires. In fact, patients presenting with alarm symptoms had even fewer persistent CeD-related symptoms on long-term GFD. The reasons for this remain speculative; for instance, in some cases, milder symptoms may have been attributable to conditions such as irritable bowel syndrome, and therefore are less responsive to gluten withdrawal. Overall, the underlying causes of persistent symptoms in patients with CeD are complex and likely often multifactorial in nature [32,33]. In terms of adherence to strict GFD, positivity for serum CeD autoantibodies or presence of comorbidities, the groups did not differ after adjustments. Overall, these results show that patients with CeD presenting with alarm symptoms did not have poorer long-term treatment outcomes than those without such symptoms.

There is a strong tendency towards a no-biopsy approach for CeD in adults, as high levels of TGA demonstrate excellent specificity [9,10,34], and avoiding biopsies could save a significant amount of resources [35]. It has been suggested that patients presenting with alarm symptoms should undergo endoscopies, as severe comorbidities could otherwise be missed [10]; however, in previous studies, alarm symptoms at CeD diagnosis have not predicted comorbidities in the upper gastrointestinal tract [2,3]. No cases of malignancy were found in these studies, but the patients had a median age of only 35 and 38 years, respectively. In the present study, two patients were diagnosed with small-bowel malignancy, both of whom experienced unintended weight loss and recurrent vomiting. In our earlier analysis of patients with CeD with a median age of 50 years, one malignancy was found in a patient presenting with dysphagia [21]. Overall, early diagnosis and strict adherence to GFD promote a favorable prognosis, while long-standing untreated CeD increases the risk of a complicated disease course [6,30,36,37]. At the same time, age is a risk factor for all-cause morbidity. Together, these factors support the idea of performing routine endoscopies in patients diagnosed later in life and in those presenting with alarm symptoms that are less typical of CeD, but further research on this is needed. In the future, comparing long-term outcomes between biopsy-diagnosed and serologically diagnosed patients is particularly important.

Altogether, the prevalence of all malignancies, including those outside the gastrointestinal tract, was low in this study, regardless of the clinical presentation at CeD diagnosis; however, it is important to note that the present study does not specify when the malignancies were diagnosed following the CeD diagnosis, nor does it indicate which predictive symptoms, if any, occurred beforehand. Further, the recruitment method might raise concerns about survivorship bias regarding malignancies. We recently compared mortality in patients with CeD with different phenotypes to that in age- and gender-matched nonceliac controls after a median follow-up of 27 years, and there was no difference between patients presenting with malabsorption – including weight loss and anaemia – and controls [38]. In addition, in another study, cancer incidence was not higher in patients with CeD presenting with malabsorption compared with the general population of the corresponding area [39]. Regardless, it is important to advise all patients with CeD to seek medical attention if symptoms persist on a strict GFD or if new symptoms and/or clinical findings emerge. Moreover, general healthcare advice on malignancies unrelated to CeD should also be a part of routine care for patients with CeD.

The main strengths of the present study were the large and representative cohort of patients with confirmed CeD diagnoses and the availability of long-term follow-up data. Furthermore, CeD-related data were obtained utilizing predesigned interviews complemented by systemically maintained patient records, and validated questionnaires were employed to assess gastrointestinal symptoms and quality of life. On the other hand, the recording of the medical data was not originally planned for the present analyses and may therefore be incomplete. In particular, CeD serology at the time of diagnosis was not available for a substantial proportion of patients. In addition, patient interviews are subject to recall bias. Further, adherence to GFD was not assessed by a dietitian, and no validated disease-specific questionnaire was used to evaluate potential CeD-related symptoms during the study visit. In addition, recruiting participants through a self-selection process may have introduced selection bias. Excluding patients in poor health and deceased patients further introduced survival bias, affecting the generalizability of the results.

### Conclusion

Despite having more advanced clinical and histological disease at diagnosis, patients with CeD presenting with alarm symptoms generally did not experience poorer long-term treatment outcomes or self-perceived health compared to those without such symptoms, although they had a higher prevalence of osteopenia or osteoporosis. This finding indicates that alarm symptoms at the time of CeD diagnosis might not always necessitate further investigations, at least in younger patients; however, the importance of careful clinical judgement and individualized follow-up remains crucial, especially for older adults and those with atypical presentations, particularly as we are moving toward less invasive methods for diagnosing CeD.

## Acknowledgements

This study was financially supported by the Competitive State Research Financing of the Expert Responsibility Area of Tampere University Hospital, the Finnish Pediatric Foundation, Päivikki and Sakari Sohlberg Foundation, Emil Aaltonen Foundation, the Academy of Finland and the Finnish Cultural Foundation.

### Conflicts of interest

K.K., K.K., and R.N. have received personal lecture fees from the Finnish Coeliac Society outside the submitted work and serve as members of the advisory committee of the Finnish Coeliac Society. For the remaining authors, there are no conflicts of interest.

## Supplementary Material
