# Supplementary material for: Presence of alarm symptoms at coeliac disease diagnosis is not associated with poorer long-term treatment outcomes
Source: Eur J Gastroenterol Hepatol. 2025 Sep 22;38(4):416–21. doi: 10.1097/MEG.0000000000003073 (PMC12935181; doi:10.1097/MEG.0000000000003073)
Supplement: Supplementary file 1 [file ejgh-38-416-s001.docx]

| Supplementary Table 1. Characteristics of 814 coeliac disease (CeD) patients with or without anaemia and with or without weight loss at the time of diagnosis | | | | | | | |
| --- | --- | --- | --- | --- | --- | --- | --- |
|  | | Anaemia  n = 257  % | No anaemia  n = 557  % | P value | Weight loss  n = 142  % | No weight loss  n = 672  % | P value |
| Females | | 86.8 | 71.5 | < 0.001 | 74.6 | 76.6 | 0.613 |
| Age, median (quartiles), yr | | 43 (35-49) | 44 (33-54) | 0.169 | 43.5 (34-52) | 43 (33-52) | 0.980 |
| Presenting symptoms | |  |  |  |  |  |  |
|  | Asymptomatic^1^ | 7.0 | 4.5 | 0.131 | 0.0 | 6.4 | **0.002*** |
|  | Gastrointestinal symptoms | 77.8 | 84.4 | **0.021*** | 88.7 | 81.0 | **0.027*** |
|  | Extraintestinal symptoms | 17.1 | 16.3 | 0.761 | 14.1 | 17.1 | 0.378 |
| Severity of symptoms | |  |  | **0.010*** |  |  | **<0.001*** |
|  | Mild | 23.1 | 29.3 |  | 0.7 | 33.4 |  |
|  | Moderate | 51.3 | 54.0 |  | 20.4 | 60.6 |  |
|  | Severe | 25.6 | 16.7 |  | 78.9 | 5.9 |  |
| Duration of symptoms | |  |  | **< 0.001*** |  |  | 0.212 |
|  | <1 yr | 12.8 | 24.6 |  | 25.5 | 19.8 |  |
|  | 1-5 yrs | 33.8 | 34.4 |  | 31.4 | 34.8 |  |
|  | 5-10 yrs | 7.3 | 11.8 |  | 13.1 | 9.7 |  |
|  | >10 yrs | 46.2 | 29.2 |  | 29.9 | 35.7 |  |
| Positive CeD serology^2,3^ | | 87.8 | 85.6 | 0.539 | 78.4 | 88.0 | **0.028*** |
| Small-bowel mucosal damage^4^ | |  |  | **< 0.001*** |  |  | **0.039*** |
|  | Normal histology^5^ | 2.7 | 3.1 |  | 2.4 | 3.2 |  |
|  | Partial villous atrophy | 21.3 | 39.4 |  | 24.4 | 35.5 |  |
|  | Subtotal or total villous atrophy | 76.9 | 57.5 |  | 73.2 | 61.5 |  |
| *P < 0.05 after adjusting for sex. ^1^Screen-detected patients in at-risk groups of CeD; ^2^Endomysial and tissue transglutaminase antibodies; Data were available from ^3^433 (53%) patients and ^4^678 (83%) patients. ^5^CeD was diagnosed on the basis of a combination of serological and histological findings and special methods, such as mucosal transglutaminase-specific IgA deposits. Values in bold face denote statistical significance. | | | | | | | |
