# Supplementary material for: Presence of alarm symptoms at coeliac disease diagnosis is not associated with poorer long-term treatment outcomes
Source: Eur J Gastroenterol Hepatol. 2025 Sep 22;38(4):416–21. doi: 10.1097/MEG.0000000000003073 (PMC12935181; doi:10.1097/MEG.0000000000003073)
Supplement: Supplementary file 2 [file ejgh-38-416-s002.docx]

| Supplementary Table 2. Characteristics of 814 coeliac disease (CeD) patients with or without anaemia and with or without weight loss on a gluten-free diet (GFD) | | | | | | | |
| --- | --- | --- | --- | --- | --- | --- | --- |
|  | | Anaemia  n=257  % | No anaemia  n=557  % | P value | Weight loss  n=142  % | No weight loss  n=672  % | P value |
| Age, median (quartiles), yr | | 54 (45-62) | 55 (44-64) | 0.520 | 57 (48-66) | 54 (44-63) | 0.030 |
| Duration of GFD, median (quartiles), yr | | 9 (5-16) | 9 (4-14) | 0.054 | 12 (6-19) | 8 (4-14) | **<0.001*** |
| Strictness of GFD | |  |  | 0.596 |  |  | 0.126 |
|  | Strict diet | 98.0 | 96.9 |  | 97.9 | 97.2 |  |
|  | Occasional lapses | 2.0 | 2.9 |  | 1.4 | 2.8 |  |
|  | Normal gluten-containing diet | 0.0 | 0.2 |  | 0.7 | 0.0 |  |
| Duodenal histology after 1 year on GFD^1^ | |  |  | 0.059 |  |  | **0.005*** |
|  | Normal histology | 51.1 | 62.1 |  | 43.3 | 61.5 |  |
|  | Partial villous atrophy | 42.0 | 33.6 |  | 47.8 | 34.1 |  |
|  | Subtotal or total villous atrophy | 6.9 | 4.3 |  | 8.9 | 4.4 |  |
| Positive endomysial antibodies | | 7.4 | 6.6 | 0.694 | 7.7 | 6.7 | 0.653 |
| Positive transglutaminase antibodies | | 11.5 | 10.8 | 0.771 | 10.6 | 11.1 | 0.884 |
| Persistent CeD-related symptoms | |  |  | 0.062 |  |  | 0.292 |
|  | No | 79.0 | 72.3 |  | 79.6 | 73.3 |  |
|  | Mild | 18.7 | 26.1 |  | 19.0 | 24.8 |  |
|  | Severe | 2.4 | 1.6 |  | 1.4 | 2.0 |  |
| Chronic comorbidities | | 90.2 | 88.7 | 0.546 | 85.9 | 89.9 | 0.167 |
|  | Gastrointestinal disease | 34.8 | 35.7 | 0.813 | 35.9 | 35.3 | 0.923 |
|  | Autoimmune disease | 41.8 | 41.8 | 1.000 | 41.5 | 41.8 | 1.000 |
|  | Musculoskeletal disease | 15.2 | 23.3 | **0.009*** | 16.9 | 21.6 | 0.255 |
|  | Osteoporosis or osteopenia | 18.4 | 8.4 | **<0.001*** | 11.3 | 11.6 | 0.908 |
|  | Any fracture | 31.8 | 25.5 | 0.063 | 29.1 | 27.1 | 0.636 |
|  | Psychiatric disease | 4.3 | 5.0 | 0.655 | 4.2 | 4.9 | 0.728 |
|  | Malignancy | 4.7 | 2.9 | 0.187 | 4.9 | 3.2 | 0.300 |
| GSRS total score, median (quartiles)^2^ | | 2.0 (1.5-2.6) | 1.9 (1.5-2.6) | 0.634 | 2.0 (1.5-2.7) | 1.9 (1.5-2.5) | 0.482 |
| PGWB total score, median PGWB total, median (quartiles)^2^ | | 106 (94-115) | 106 (94-115) | 0.977 | 106 (93-116) | 106 (95-115) | 0.893 |
| * P < 0.05 after adjusting for sex and duration of GFD. ^1^Data available from 462 (57%) and ^2^590 (73%) of patients. GSRS, Gastrointestinal Symptom Rating Scale; PGWB, Psychological General Well-Being. Values in bold face denote statistical significance. | | | | | | | |
